# 3,3-Bis(4-bromo­phenyl­sulfan­yl)-1-methyl­piperidin-2-one

**DOI:** 10.1107/S1600536813006995

**Published:** 2013-03-20

**Authors:** Julio Zukerman-Schpector, Paulo R. Olivato, Carlos R. Cerqueira Jr, Bruna Contieri, Seik Weng Ng, Edward R. T. Tiekink

**Affiliations:** a Departmento de Química, Universidade Federal de São Carlos, CP 676, 13565-905 São Carlos-SP, Brazil; b Chemistry Institute, University of São Paulo, 05508-000 São Paulo, SP, Brazil; c Department of Chemistry, University of Malaya, 50603 Kuala Lumpur, Malaysia; d Chemistry Department, Faculty of Science, King Abdulaziz University, PO Box 80203 Jeddah, Saudi Arabia

## Abstract

In the title compound, C_18_H_17_Br_2_NOS_2_, the conformation of the piperidin-2-one ring is based on a half-chair with the methyl­ene C atom diagonally opposite the N atom being 0.649 (3) Å above the plane of the remaining five atoms (r.m.s. deviation = 0.1205 Å). The S atoms occupy axial and bis­ectional positions, and the dihedral angle between the benzene rings of 59.95 (11)° indicates a splayed disposition. Helical supra­molecular chains along the *b* axis sustained by C—H⋯O inter­actions is the major feature of the crystal packing. The chains are connected into a three-dimensional architecture by C—H⋯Br and C—H⋯π inter­actions.

## Related literature

For background to the chemistry and structures of β-thio-carbonyl compounds, see: Zukerman-Schpector *et al.* (2009 ▶); Vinhato (2007 ▶); Vinhato *et al.* (2011 ▶); Olivato *et al.* (2012 ▶, 2013 ▶). For the synthesis, see: Olivato *et al.* (2013 ▶). For ring conformational analysis, see: Cremer & Pople (1975 ▶).
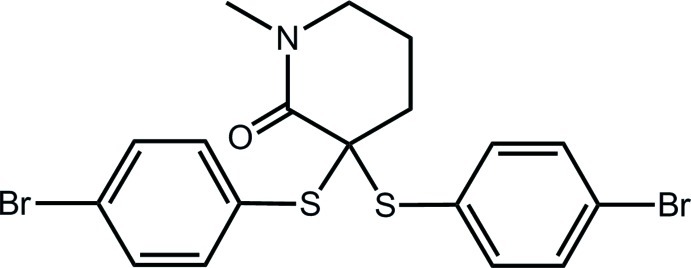


## Experimental

### 

#### Crystal data

C_18_H_17_Br_2_NOS_2_
*M* *_r_* = 487.27
Monoclinic, 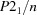

*a* = 7.8777 (1) Å
*b* = 9.6481 (1) Å
*c* = 24.6757 (3) Å
β = 93.190 (1)°
*V* = 1872.57 (4) Å^3^
*Z* = 4
Cu *K*α radiation
μ = 7.61 mm^−1^
*T* = 100 K
0.25 × 0.25 × 0.05 mm

#### Data collection

Agilent SuperNova (Dual, Cu at zero, Atlas) diffractometer
Absorption correction: multi-scan (*CrysAlis PRO*; Agilent, 2011 ▶) *T*_min_ = 0.298, *T*_max_ = 1.000
18656 measured reflections
3916 independent reflections
3715 reflections with *I* > 2σ(*I*)
*R*_int_ = 0.038

#### Refinement

*R*[*F* ^2^ > 2σ(*F* ^2^)] = 0.029
*wR*(*F* ^2^) = 0.077
*S* = 1.10
3916 reflections
218 parameters
H-atom parameters constrained
Δρ_max_ = 0.71 e Å^−3^
Δρ_min_ = −1.26 e Å^−3^

### 

Data collection: *CrysAlis PRO* (Agilent, 2011 ▶); cell refinement: *CrysAlis PRO*; data reduction: *CrysAlis PRO*; program(s) used to solve structure: *SIR92* (Altomare *et al.*, 1999 ▶); program(s) used to refine structure: *SHELXL97* (Sheldrick, 2008 ▶); molecular graphics: *ORTEP-3 for Windows* (Farrugia, 2012 ▶) and *DIAMOND* (Brandenburg, 2006 ▶); software used to prepare material for publication: *publCIF* (Westrip, 2010 ▶).

## Supplementary Material

Crystal structure: contains datablock(s) global, I. DOI: 10.1107/S1600536813006995/hg5299sup1.cif

Structure factors: contains datablock(s) I. DOI: 10.1107/S1600536813006995/hg5299Isup2.hkl

Supplementary material file. DOI: 10.1107/S1600536813006995/hg5299Isup3.cml

Additional supplementary materials:  crystallographic information; 3D view; checkCIF report

## Figures and Tables

**Table 1:** Hydrogen-bond geometry (Å, °) *Cg*1 is the centroid of the C7–C12 ring.

*D*—H⋯*A*	*D*—H	H⋯*A*	*D*⋯*A*	*D*—H⋯*A*
C9—H9⋯Br2^i^	0.95	2.87	3.744 (2)	154
C11—H11⋯O1^ii^	0.95	2.27	3.195 (3)	163
C1—H1*B*⋯*Cg*1^i^	0.98	2.86	3.660 (3)	139

Symmetry codes: (i) 
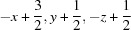
; (ii) 
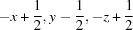
.

**Fig. 1.**
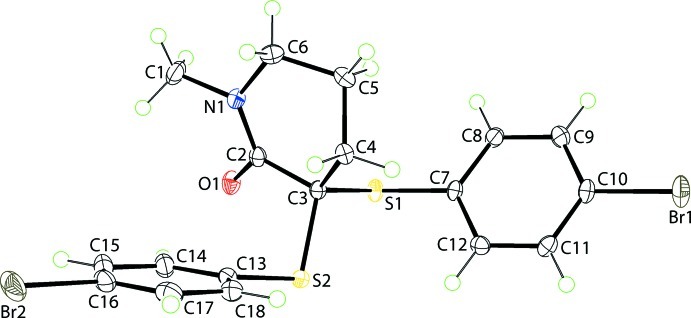
Molecular structure of (I) showing atom labelling and displacement ellipsoids at the 50% probability level.

**Fig. 2.**
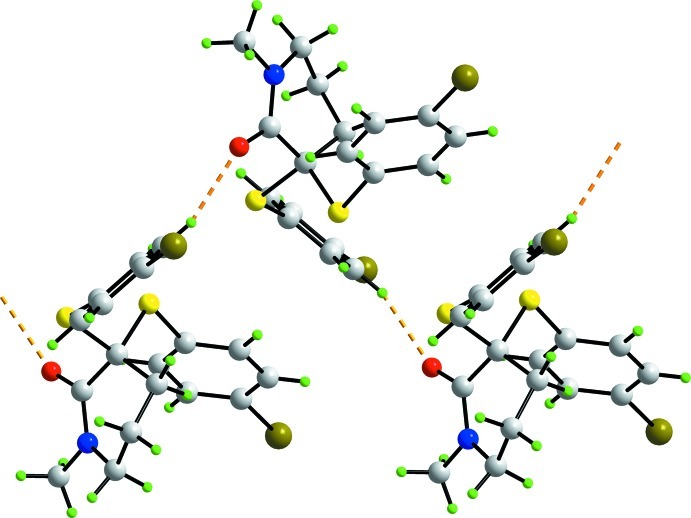
Helical supramolecular chain along the b axis sustained by C—H···.O interactions (blue dashed lines).

**Fig. 3.**
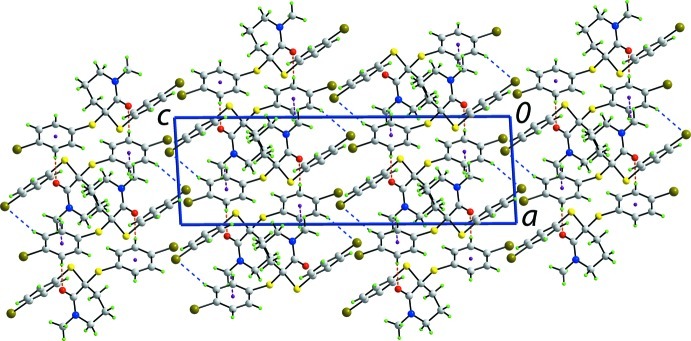
View in projection down the b axis of the unit-cell contents. The C—H···O, C—H···Br and C—H···π interactions are shown as blue, orange and purple dashed lines, respectively.

## supplementary crystallographic information

### Comment

The title compound (I), Fig. 1, was studied as a part of an on-going investigation of conformational and electronic interactions in β-thio-carbonyl compounds, *e.g. N*,*N*-diethyl-2-[(4'-substituted) phenylthio]acetamides, *N*,*N*-diethyl-2-[(4'-substituted) phenylsulfonyl]acetamides and 3,3-bis[(4'-substituted phenylsulfany)]-1-methyl-2-piperidinones using spectroscopic, theoretical and X-ray diffraction methods (Vinhato, 2007; Zukerman-Schpector *et al.*, 2009; Vinhato *et al.*, 2011; Olivato *et al.*, 2012; Olivato *et al.*, 2013).

In (I), the conformation of the six-membered piperidin-2-one ring is highly distorted with the best description being one based on a half-chair with the C4 atom lying 0.649 (3) Å above the plane of the remaining five atoms (r.m.s. deviation = 0.1205 Å), with puckering parameters: q_2_ = 0.463 (2) Å and q_3_ = 0.275 (2) Å, and amplitudes: Q = 0.539 (2) Å, θ = 59.4 (2)° and φ_2_ = 214.7 (3)° (Cremer & Pople, 1975). The carbonyl-O1 and methyl-C1 atom occupy equatorial positions with respect to the piperidinyl ring while the S1 and S2 atoms are axial and bisectional, respectively. The dihedral angle between the benzene rings is 59.95 (11)°, indicating a splayed disposition.

The crystal packing features helical supramolecular chains along the *b* axis sustained by rather strong C—H···O interactions, Fig. 2 and Table 1. These are consolidated into a three-dimensional architecture by C—H···Br and C—H···π interactions, Fig. 3 and Table 1.

### Experimental

The preparation of the title compound was recently described (Olivato *et al.*, 2013). Suitable crystals were obtained by vapour diffusion of *n*-hexane into a chloroform solution at 283 K.; *M*.pt: 383–385 K.

### Refinement

All H atoms were included in the riding-model approximation with C—H = 0.95–0.99 Å, and with *U*_iso_(H) = 1.5*U*_eq_(methyl-C) and 1.2*U*_eq_(remaining-C). The maximum and minimum residual electron density peaks of 0.71 and -1.26 e Å^-3^, respectively, were located 0.77 and 0.72 Å from the Br2 atom.

### Crystal data

**Table:**

C_18_H_17_Br_2_NOS_2_	*F*(000) = 968
*M**_r_* = 487.27	*D*_x_ = 1.728 Mg m^−^^3^
Monoclinic, *P*2_1_/*n*	Cu *K*α radiation, λ = 1.5418 Å
Hall symbol: -P 2yn	Cell parameters from 10597 reflections
*a* = 7.8777 (1) Å	θ = 3.6–76.5°
*b* = 9.6481 (1) Å	µ = 7.61 mm^−^^1^
*c* = 24.6757 (3) Å	*T* = 100 K
β = 93.190 (1)°	Prism, colourless
*V* = 1872.57 (4) Å^3^	0.25 × 0.25 × 0.05 mm
*Z* = 4	

### Data collection

**Table:**

Agilent SuperNova (Dual, Cu at zero, Atlas) diffractometer	3916 independent reflections
Radiation source: SuperNova (Cu) X-ray Source	3715 reflections with *I* > 2σ(*I*)
Mirror monochromator	*R*_int_ = 0.038
Detector resolution: 10.4041 pixels mm^-1^	θ_max_ = 76.7°, θ_min_ = 3.6°
ω scans	*h* = −8→9
Absorption correction: multi-scan (*CrysAlis PRO*; Agilent, 2011)	*k* = −11→12
*T*_min_ = 0.298, *T*_max_ = 1.000	*l* = −31→30
18656 measured reflections	

### Refinement

**Table:**

Refinement on *F*^2^	Primary atom site location: structure-invariant direct methods
Least-squares matrix: full	Secondary atom site location: difference Fourier map
*R*[*F*^2^ > 2σ(*F*^2^)] = 0.029	Hydrogen site location: inferred from neighbouring sites
*wR*(*F*^2^) = 0.077	H-atom parameters constrained
*S* = 1.10	*w* = 1/[σ^2^(*F*_o_^2^) + (0.0402*P*)^2^ + 1.5955*P*] where *P* = (*F*_o_^2^ + 2*F*_c_^2^)/3
3916 reflections	(Δ/σ)_max_ < 0.001
218 parameters	Δρ_max_ = 0.71 e Å^−^^3^
0 restraints	Δρ_min_ = −1.26 e Å^−^^3^

### Special details

**Table:**

Geometry. All s.u.'s (except the s.u. in the dihedral angle between two l.s. planes) are estimated using the full covariance matrix. The cell s.u.'s are taken into account individually in the estimation of s.u.'s in distances, angles and torsion angles; correlations between s.u.'s in cell parameters are only used when they are defined by crystal symmetry. An approximate (isotropic) treatment of cell s.u.'s is used for estimating s.u.'s involving l.s. planes.
Refinement. Refinement of *F*^2^ against ALL reflections. The weighted *R*-factor *wR* and goodness of fit *S* are based on *F*^2^, conventional *R*-factors *R* are based on *F*, with *F* set to zero for negative *F*^2^. The threshold expression of *F*^2^ > σ(*F*^2^) is used only for calculating *R*-factors(gt) *etc*. and is not relevant to the choice of reflections for refinement. *R*-factors based on *F*^2^ are statistically about twice as large as those based on *F*, and *R*- factors based on ALL data will be even larger.

### Fractional atomic coordinates and isotropic or equivalent isotropic displacement parameters (Å^2^)

**Table:**

	*x*	*y*	*z*	*U*_iso_*/*U*_eq_	
Br1	0.20835 (4)	0.58290 (3)	0.025429 (10)	0.02829 (9)	
Br2	0.82389 (3)	0.31232 (3)	0.521415 (11)	0.03241 (9)	
S1	0.43546 (6)	0.86583 (5)	0.25378 (2)	0.01528 (11)	
S2	0.39439 (6)	0.64897 (5)	0.33476 (2)	0.01610 (11)	
O1	0.6089 (2)	0.90775 (16)	0.36438 (6)	0.0190 (3)	
N1	0.8407 (2)	0.81429 (19)	0.32947 (7)	0.0166 (4)	
C1	0.9475 (3)	0.8914 (3)	0.36927 (10)	0.0229 (5)	
H1A	0.9052	0.8781	0.4055	0.034*	
H1B	0.9443	0.9903	0.3601	0.034*	
H1C	1.0648	0.8577	0.3689	0.034*	
C2	0.6716 (3)	0.8271 (2)	0.33253 (8)	0.0144 (4)	
C3	0.5560 (3)	0.7345 (2)	0.29522 (8)	0.0132 (4)	
C4	0.6530 (3)	0.6307 (2)	0.26235 (8)	0.0143 (4)	
H4A	0.5778	0.5947	0.2321	0.017*	
H4B	0.6897	0.5516	0.2857	0.017*	
C5	0.8078 (3)	0.6999 (2)	0.23983 (9)	0.0168 (4)	
H5A	0.8652	0.6347	0.2159	0.020*	
H5B	0.7722	0.7825	0.2182	0.020*	
C6	0.9284 (3)	0.7424 (2)	0.28685 (9)	0.0195 (4)	
H6A	0.9853	0.6588	0.3024	0.023*	
H6B	1.0172	0.8040	0.2733	0.023*	
C7	0.3782 (3)	0.7802 (2)	0.19177 (8)	0.0138 (4)	
C8	0.4438 (3)	0.8300 (2)	0.14454 (9)	0.0183 (4)	
H8	0.5235	0.9040	0.1463	0.022*	
C9	0.3934 (3)	0.7720 (2)	0.09462 (9)	0.0208 (4)	
H9	0.4372	0.8061	0.0621	0.025*	
C10	0.2776 (3)	0.6631 (2)	0.09327 (9)	0.0171 (4)	
C11	0.2116 (3)	0.6111 (2)	0.13980 (9)	0.0167 (4)	
H11	0.1333	0.5361	0.1379	0.020*	
C12	0.2618 (3)	0.6707 (2)	0.18945 (9)	0.0154 (4)	
H12	0.2168	0.6369	0.2219	0.018*	
C13	0.5212 (3)	0.5553 (2)	0.38409 (9)	0.0163 (4)	
C14	0.5898 (3)	0.6240 (2)	0.42994 (9)	0.0208 (4)	
H14	0.5735	0.7211	0.4336	0.025*	
C15	0.6815 (3)	0.5517 (3)	0.47026 (9)	0.0239 (5)	
H15	0.7290	0.5986	0.5014	0.029*	
C16	0.7031 (3)	0.4095 (3)	0.46455 (10)	0.0222 (5)	
C17	0.6353 (3)	0.3388 (2)	0.41976 (10)	0.0228 (5)	
H17	0.6504	0.2415	0.4166	0.027*	
C18	0.5445 (3)	0.4126 (2)	0.37925 (9)	0.0193 (4)	
H18	0.4980	0.3654	0.3481	0.023*	

### Atomic displacement parameters (Å^2^)

**Table:**

	*U*^11^	*U*^22^	*U*^33^	*U*^12^	*U*^13^	*U*^23^
Br1	0.04084 (17)	0.02690 (14)	0.01606 (13)	−0.00348 (10)	−0.00800 (10)	−0.00344 (9)
Br2	0.03033 (15)	0.04426 (17)	0.02278 (14)	0.01094 (11)	0.00269 (10)	0.01732 (11)
S1	0.0192 (2)	0.0127 (2)	0.0134 (2)	0.00287 (17)	−0.00357 (18)	−0.00037 (17)
S2	0.0128 (2)	0.0221 (2)	0.0133 (2)	−0.00130 (18)	−0.00071 (17)	0.00314 (18)
O1	0.0211 (7)	0.0191 (7)	0.0165 (8)	0.0035 (6)	−0.0020 (6)	−0.0051 (6)
N1	0.0158 (9)	0.0185 (9)	0.0153 (9)	−0.0012 (7)	−0.0013 (7)	−0.0003 (7)
C1	0.0186 (10)	0.0263 (11)	0.0230 (11)	−0.0046 (9)	−0.0066 (8)	−0.0012 (9)
C2	0.0170 (10)	0.0129 (9)	0.0131 (9)	0.0011 (7)	−0.0013 (7)	0.0025 (7)
C3	0.0143 (9)	0.0127 (9)	0.0123 (9)	0.0005 (7)	−0.0006 (7)	0.0004 (7)
C4	0.0176 (9)	0.0122 (9)	0.0130 (9)	0.0014 (7)	0.0004 (7)	−0.0002 (7)
C5	0.0198 (10)	0.0161 (9)	0.0149 (10)	0.0028 (8)	0.0048 (8)	0.0016 (7)
C6	0.0141 (10)	0.0211 (10)	0.0235 (11)	0.0013 (8)	0.0032 (8)	−0.0003 (8)
C7	0.0141 (9)	0.0141 (9)	0.0128 (9)	0.0018 (7)	−0.0033 (7)	0.0002 (7)
C8	0.0177 (10)	0.0196 (10)	0.0170 (10)	−0.0052 (8)	−0.0032 (8)	0.0050 (8)
C9	0.0226 (11)	0.0263 (11)	0.0134 (10)	−0.0029 (9)	−0.0008 (8)	0.0051 (8)
C10	0.0189 (10)	0.0178 (10)	0.0140 (10)	0.0020 (8)	−0.0054 (8)	−0.0007 (8)
C11	0.0150 (9)	0.0152 (9)	0.0193 (10)	−0.0001 (8)	−0.0035 (8)	0.0008 (8)
C12	0.0151 (9)	0.0159 (9)	0.0152 (10)	0.0015 (7)	0.0010 (7)	0.0035 (7)
C13	0.0135 (9)	0.0214 (10)	0.0139 (10)	−0.0015 (8)	0.0006 (7)	0.0039 (8)
C14	0.0242 (11)	0.0221 (11)	0.0158 (10)	−0.0019 (9)	−0.0014 (8)	0.0017 (8)
C15	0.0281 (12)	0.0282 (12)	0.0150 (10)	−0.0042 (9)	−0.0031 (9)	0.0023 (9)
C16	0.0181 (10)	0.0298 (12)	0.0187 (11)	0.0019 (8)	0.0005 (8)	0.0119 (9)
C17	0.0227 (11)	0.0215 (11)	0.0247 (12)	0.0012 (9)	0.0052 (9)	0.0060 (9)
C18	0.0192 (10)	0.0203 (10)	0.0183 (10)	−0.0035 (8)	0.0018 (8)	0.0007 (8)

### Geometric parameters (Å, º)

**Table:**

Br1—C10	1.897 (2)	C6—H6B	0.9900
Br2—C16	1.899 (2)	C7—C8	1.387 (3)
S1—C7	1.775 (2)	C7—C12	1.398 (3)
S1—C3	1.856 (2)	C8—C9	1.391 (3)
S2—C13	1.778 (2)	C8—H8	0.9500
S2—C3	1.842 (2)	C9—C10	1.390 (3)
O1—C2	1.229 (3)	C9—H9	0.9500
N1—C2	1.344 (3)	C10—C11	1.381 (3)
N1—C1	1.461 (3)	C11—C12	1.391 (3)
N1—C6	1.465 (3)	C11—H11	0.9500
C1—H1A	0.9800	C12—H12	0.9500
C1—H1B	0.9800	C13—C14	1.394 (3)
C1—H1C	0.9800	C13—C18	1.396 (3)
C2—C3	1.543 (3)	C14—C15	1.385 (3)
C3—C4	1.521 (3)	C14—H14	0.9500
C4—C5	1.522 (3)	C15—C16	1.391 (3)
C4—H4A	0.9900	C15—H15	0.9500
C4—H4B	0.9900	C16—C17	1.380 (4)
C5—C6	1.515 (3)	C17—C18	1.392 (3)
C5—H5A	0.9900	C17—H17	0.9500
C5—H5B	0.9900	C18—H18	0.9500
C6—H6A	0.9900		
			
C7—S1—C3	104.77 (9)	H6A—C6—H6B	107.9
C13—S2—C3	102.22 (9)	C8—C7—C12	120.11 (19)
C2—N1—C1	116.82 (18)	C8—C7—S1	118.33 (16)
C2—N1—C6	126.33 (18)	C12—C7—S1	121.44 (16)
C1—N1—C6	116.53 (18)	C7—C8—C9	120.2 (2)
N1—C1—H1A	109.5	C7—C8—H8	119.9
N1—C1—H1B	109.5	C9—C8—H8	119.9
H1A—C1—H1B	109.5	C10—C9—C8	118.7 (2)
N1—C1—H1C	109.5	C10—C9—H9	120.7
H1A—C1—H1C	109.5	C8—C9—H9	120.7
H1B—C1—H1C	109.5	C11—C10—C9	122.1 (2)
O1—C2—N1	121.9 (2)	C11—C10—Br1	118.85 (16)
O1—C2—C3	120.20 (18)	C9—C10—Br1	119.05 (17)
N1—C2—C3	117.86 (18)	C10—C11—C12	118.72 (19)
C4—C3—C2	113.70 (17)	C10—C11—H11	120.6
C4—C3—S2	111.69 (14)	C12—C11—H11	120.6
C2—C3—S2	110.22 (14)	C11—C12—C7	120.1 (2)
C4—C3—S1	114.37 (14)	C11—C12—H12	119.9
C2—C3—S1	101.59 (13)	C7—C12—H12	119.9
S2—C3—S1	104.48 (10)	C14—C13—C18	119.5 (2)
C3—C4—C5	109.99 (17)	C14—C13—S2	119.46 (17)
C3—C4—H4A	109.7	C18—C13—S2	120.96 (17)
C5—C4—H4A	109.7	C15—C14—C13	120.4 (2)
C3—C4—H4B	109.7	C15—C14—H14	119.8
C5—C4—H4B	109.7	C13—C14—H14	119.8
H4A—C4—H4B	108.2	C14—C15—C16	119.1 (2)
C6—C5—C4	108.68 (17)	C14—C15—H15	120.5
C6—C5—H5A	110.0	C16—C15—H15	120.5
C4—C5—H5A	110.0	C17—C16—C15	121.6 (2)
C6—C5—H5B	110.0	C17—C16—Br2	120.25 (18)
C4—C5—H5B	110.0	C15—C16—Br2	118.10 (18)
H5A—C5—H5B	108.3	C16—C17—C18	118.9 (2)
N1—C6—C5	112.15 (17)	C16—C17—H17	120.6
N1—C6—H6A	109.2	C18—C17—H17	120.6
C5—C6—H6A	109.2	C17—C18—C13	120.5 (2)
N1—C6—H6B	109.2	C17—C18—H18	119.7
C5—C6—H6B	109.2	C13—C18—H18	119.7
			
C1—N1—C2—O1	5.5 (3)	C3—S1—C7—C12	67.81 (18)
C6—N1—C2—O1	−167.7 (2)	C12—C7—C8—C9	0.4 (3)
C1—N1—C2—C3	−173.40 (18)	S1—C7—C8—C9	−175.67 (17)
C6—N1—C2—C3	13.4 (3)	C7—C8—C9—C10	−0.5 (3)
O1—C2—C3—C4	−174.26 (18)	C8—C9—C10—C11	0.0 (3)
N1—C2—C3—C4	4.6 (3)	C8—C9—C10—Br1	−179.85 (17)
O1—C2—C3—S2	−48.0 (2)	C9—C10—C11—C12	0.6 (3)
N1—C2—C3—S2	130.94 (17)	Br1—C10—C11—C12	−179.60 (15)
O1—C2—C3—S1	62.4 (2)	C10—C11—C12—C7	−0.6 (3)
N1—C2—C3—S1	−118.72 (17)	C8—C7—C12—C11	0.1 (3)
C13—S2—C3—C4	68.86 (16)	S1—C7—C12—C11	176.10 (16)
C13—S2—C3—C2	−58.56 (15)	C3—S2—C13—C14	80.85 (19)
C13—S2—C3—S1	−166.99 (10)	C3—S2—C13—C18	−103.11 (18)
C7—S1—C3—C4	30.32 (17)	C18—C13—C14—C15	0.5 (3)
C7—S1—C3—C2	153.23 (13)	S2—C13—C14—C15	176.61 (18)
C7—S1—C3—S2	−92.10 (11)	C13—C14—C15—C16	−0.5 (4)
C2—C3—C4—C5	−42.8 (2)	C14—C15—C16—C17	0.0 (4)
S2—C3—C4—C5	−168.28 (14)	C14—C15—C16—Br2	−178.26 (18)
S1—C3—C4—C5	73.31 (19)	C15—C16—C17—C18	0.5 (4)
C3—C4—C5—C6	64.5 (2)	Br2—C16—C17—C18	178.74 (17)
C2—N1—C6—C5	9.0 (3)	C16—C17—C18—C13	−0.5 (3)
C1—N1—C6—C5	−164.26 (18)	C14—C13—C18—C17	0.0 (3)
C4—C5—C6—N1	−47.3 (2)	S2—C13—C18—C17	−176.02 (17)
C3—S1—C7—C8	−116.16 (17)		

### Hydrogen-bond geometry (Å, º)

Cg1 is the centroid of the C7–C12 ring.

**Table:**

*D*—H···*A*	*D*—H	H···*A*	*D*···*A*	*D*—H···*A*
C9—H9···Br2^i^	0.95	2.87	3.744 (2)	154
C11—H11···O1^ii^	0.95	2.27	3.195 (3)	163
C1—H1*B*···*Cg*1^i^	0.98	2.86	3.660 (3)	139

Symmetry codes: (i) −*x*+3/2, *y*+1/2, −*z*+1/2; (ii) −*x*+1/2, *y*−1/2, −*z*+1/2.
